# Identifying subtypes and developing prognostic models based on N6-methyladenosine and immune microenvironment related genes in breast cancer

**DOI:** 10.1038/s41598-024-67477-w

**Published:** 2024-07-18

**Authors:** Lizhao Wang, Jianpeng Li, Nan Mei, Heyan Chen, Ligang Niu, Jianjun He, Ru Wang

**Affiliations:** 1 Department of Breast Surgery, The First Affiliated Hospital of Xi’an Jiaotong University, 277 West Yanta Road, Xi’an, 710061 Shaanxi China; 2 Department of Urology, The First Affiliated Hospital of Xi’an Jiaotong University, Xi’an, 710061 Shaanxi China; 3 Department of Hematology, The First Affiliated Hospital of Xi’an Jiaotong University, Xi’an, 710061 Shaanxi China

**Keywords:** Breast cancer, N6-methyladenosine, Immune microenvironment, Gene signature, Prognosis, Cancer, Breast cancer, Cancer microenvironment, Cancer prevention

## Abstract

Breast cancer (BC) is the most prevalent cancer in women globally. The tumor microenvironment (TME), comprising epithelial tumor cells and stromal elements, is vital for breast tumor development. N6-methyladenosine (m6A) modification plays a key role in RNA metabolism, influencing its various aspects such as stability and translation. There is a notable link between m6A methylation and immune cells in the TME, although this relationship is complex and not fully deciphered. In this research, BC expression and clinicopathological data from TCGA were scrutinized to assess expression profiles, mutations, and CNVs of 31 m6A genes and immune microenvironment-related genes, examining their correlations, functions, and prognostic impacts. Lasso and Cox regression identified prognostic genes for constructing a nomogram. Single-cell analyses mapped the distribution and patterns of these genes in BC cell development. We investigated associations between gene-derived risk scores and factors like immune infiltration, TME, checkpoints, TMB, CSC indices, and drug response. As a complement to computational analyses, in vitro experiments were conducted to confirm these expression patterns. We included 31 m6A regulatory genes and discovered a correlation between these genes and the extent of immune cell infiltration. Subsequently, a 7-gene risk score was generated, encompassing HSPA2, TAP1, ULBP2, CXCL1, RBP1, STC2, and FLT3. It was observed that the low-risk group exhibited better overall survival (OS) in BC, with higher immune scores but lower tumor mutational burden (TMB) and cancer stem cell (CSC) indices, as well as lower IC50 values for commonly used drugs. To enhance clinical applicability, age and stage were incorporated into the risk score, and a more comprehensive nomogram was constructed to predict OS. This nomogram was validated and demonstrated good predictive performance, with area under the curve (AUC) values for 1-year, 3-year, and 5-year OS being 0.848, 0.807, and 0.759, respectively. Our findings highlight the profound impact of prognostic-related genes on BC immune response and prognostic outcomes, suggesting that modulation of the m6A-immune pathway could offer new avenues for personalized BC treatment and potentially improve clinical outcomes.

## Introduction

Breast cancer (BC) is the most common malignant tumor among women worldwide. Approximately one in every eight women globally is diagnosed with BC ranking it as the second leading cause of death among female cancers^1–3^. This is a complex and diverse disease characterized by the uncontrolled proliferation and growth of abnormal cells in breast tissue. Currently, the main clinical strategies for treating BC include mastectomy, radiotherapy, chemotherapy, endocrine therapy, and targeted therapies such as immune checkpoint inhibitors (ICIs) and antibody drug conjugates (ADCs)^4–7^.

In cancer research, the “Tumor Microenvironment” (TME) is recognized as a critical factor in breast tumor progression. It encompasses a complex interplay of epithelial tumor cells, immune cells like regulatory T cells (Tregs), myeloid-derived suppressor cells, and B cells, along with extracellular matrix, cancer-associated fibroblasts, blood vessels, and adipocytes. This intricate network is essential for understanding and combating breast cancer^8^. Given the integral role of the Tumor Microenvironment (TME) in cancer study, immunotherapy, particularly with immune checkpoint inhibitors (ICIs), is gaining prominence in clinical cancer treatment for its notable therapeutic benefits^9,10^. The emergence of ICIs represents a therapeutic revolution of the past decade, with immunotherapy being an effective treatment strategy for a variety of tumors. The mechanism of action of ICIs is based on the activation of the immune system, capable of modulating T lymphocytes and targeting immune checkpoints, such as Programmed Cell Death Protein 1 (PD-1), Programmed Cell Death Ligand 1 (PD-L1), and Cytotoxic T-Lymphocyte-Associated Protein 4 (CTLA-4). As monotherapy or in combination with other anticancer drugs, ICIs have achieved unprecedented effects in melanoma, renal cell carcinoma, non-small cell lung cancer, hepatocellular carcinoma, colorectal cancer, urothelial carcinoma, and breast cancer^11–14^. This finding broadens the prospects for the application of immunotherapy in BC treatment. However, not all patients exhibit a favorable response to this type of immunotherapy^15–17^. This might be attributed to the fact that BC is a highly heterogeneous malignant tumor, displaying significant morphological and molecular heterogeneity both among tumors and even within individual tumor cells^18^. BC cells can evade immune surveillance within the TME and induce suppression of the anti-tumor immune system, leading to drug resistance and BC recurrence^19^. Moreover, ICIs may lead to unique and often specific adverse events, which are believed to be determined by an overactive, unchecked T-cell-mediated immune response, potentially leading to a reduction in their therapeutic effects^12^. Despite the astonishing development pace of ICIs at therapeutic sites, the development of biomarkers that can continuously assist in the decision-making process for ICIs has been relatively slow. There are several factors contributing to the slow development of ICI biomarkers, including the need for tissue samples and complex platforms. Furthermore, tissue biomarkers may not reflect the status of the immune system, which is the main driving factor for the efficacy of ICIs^20,21^. A new biomarker is urgently needed to fully characterize the TME and identify specific immune profiles in BC. This could help predict responses to immunotherapy, guiding personalized treatment strategies and optimizing outcomes for patients with diverse risk profiles. The search for such a biomarker has become a pivotal focus in recent breast cancer research.

Post-transcriptional regulation of the transcriptome is a crucial biological process, and to date, over 170 RNA modifications have been discovered in eukaryotes. These include 5-methylcytosine (m5C), N6-methyladenosine (m6A), and N1-methyladenosine (m1A)^22,23^. N6-methyladenosine modification is a dynamic process involved in post-transcriptional RNA modifications, which influences nearly all aspects of RNA metabolism including RNA translocation, splicing, stability, and translation. The m6A modification is mediated by three types of m6A regulatory factors, including m6A-binding proteins (readers), demethylases (erasers), and methyltransferases (writers)^24,25^. In the realm of cellular modification processes, m6A RNA methylation stands out as a prevalent feature in various RNA types such as mRNA, lncRNA, and miRNA. This modification is recognized as a key and widespread internal alteration in the cells of eukaryotic organisms^26^. Current research highlights a strong link between m6A regulatory elements and their roles in both promoting and inhibiting tumor growth. These roles encompass critical activities like cell growth, tumor formation, spread of cancer cells, and various other tumor-specific characteristics^27–29^. Additionally, recent investigations have shed light on the vital influence of m6A in the evolution of tumors and in altering the TME^30^. Furthermore, there is compelling evidence of a notable relationship between the immune cells present in the TME and m6A methylation, a connection that extends beyond mere RNA degradation^31^. Current studies are also uncovering how m6A modifications are instrumental in shaping the interactions between cancer cells and their surrounding environment, impacting the progression of the tumor and the immune reactions^32,33^. Consequently, it is essential to delve deeper into the exploration and study of the connections and control mechanisms between immune cells within the tumor microenvironment and m6A modifications to gain a better understanding of these complex interactions.

In this study, we identified differentially expressed genes (DEGs) based on three m6A subtypes. Using these DEGs along with immune-related genes, we constructed an m6A-associated immune prognostic risk model to evaluate the prognosis of BC. Furthermore, we analyzed gene mutations related to m6A, biological processes, immune landscapes, and drug sensitivity in BC.

## Methods

### Breast cancer data source and pre-processing

**Figure 1:**
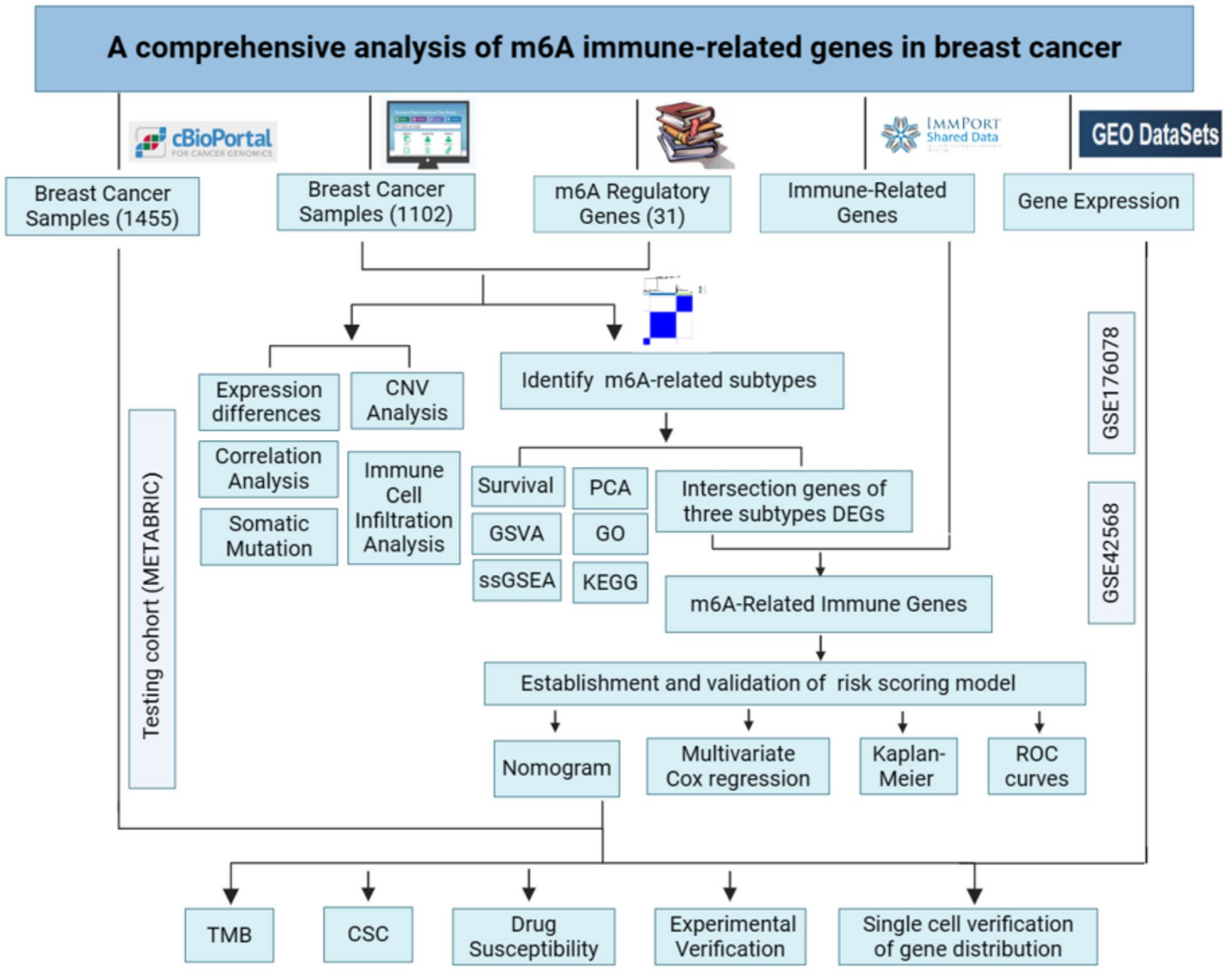
Flow chart of the study design.

The process of this study is shown in Fig. 1. We obtained mRNA expression profiles, mutations, copy number variations (CNV), and related clinicopathological data of BC samples from The Cancer Genome Atlas (TCGA) database (https://portal.gdc.cancer.gov/). A total of 1102 cases with follow-up information and complete clinicopathological data were selected from TCGA database and 1455 breast cancer samples with complete prognostic information were obtained in the METABRIC cohort from cBioportal (www. cbioportal.org) for verification. Additionally, gene expression data were downloaded from the Gene Expression Omnibus (GEO) databases (https://www.ncbi.nlm.nih.gov/geo/).

### M6A regulatory gene mutations, CNVs, and consensus clustering analysis

In accordance with previous literature, we identified 31 m6A regulatory genes, the details of which are summarized in Table S1. The positions of mutations and copy number variations (CNVs) in m6A-related genes across 23 chromosomes were visualized using the R packages “maftools” and “RCircos”. Utilizing these genes, we conducted consensus clustering analysis and categorized the samples into three distinct subtypes of m6A gene regulation using the “ConsensusClusterPlus” R package. To evaluate the overall survival (OS) among different subtypes, we employed the R packages “survival” and “survminer”. Further analysis of differentially expressed genes (DEGs) across these subtypes was conducted using the “Limma” R package. Additionally, we explored the associations between different subtypes and clinicopathological features, such as age and TNM staging, using the “pheatmap” R package. This comprehensive approach allowed us to delineate the molecular heterogeneity of BC and its implications for patient prognosis and treatment strategies.

### Immunological scoring and gene set enrichment analysis

We employed Gene Set Variation Analysis (GSVA) to examine the differences in biological processes between various m6A regulatory gene subtypes. Additionally, the present study employed the single-sample Gene Set Enrichment Analysis (ssGSEA) algorithm to evaluate the levels of immune cell infiltration in different m6A regulatory gene subtypes. Furthermore, Principal Component Analysis (PCA) was applied to classify breast cancer samples. Additionally, Gene Ontology (GO) analysis and Kyoto Encyclopedia of Genes and Genomes (KEGG) pathway analysis were conducted using R packages such as “GSEABase,” “clusterProfiler,” “GSVA,” “limma,” “org.Hs.e.g.db,” and “enrichplot” to identify biological processes associated with m6A regulatory genes.

### Construction of the m6A-related immune scoring system and prognostic risk model

We cross-analyzed the DEGs of the three subtypes previously obtained with immune-related genes to identify overlapping candidate genes, designated as our m6A immune-related genes sourced from the ImmPort shared data website, saved as Table S2. Subsequently, univariate Cox regression analysis was used to select m6A-related immune genes, with a p-value threshold of less than 0.05. LASSO regression was then applied to further refine the selection of m6A-immune related genes for subsequent analysis, identifying genes to construct the m6A Immune Score. Multivariate Cox regression was then performed on the selected genes. The screened genes are used to calculate the risk score. Use the following formula to generate a prognostic risk score for each patient:

Risk score = coefficient 1 × value l + coefficient n × value n,

Where the value is the relative expression of each selected gene's z-score conversion. Using the “rms” R package, we constructed nomograms incorporating risk scores and clinicopathological features. The discriminative ability of the model was evaluated using the Area Under the Curve (AUC) of transient ROC curves. Patients were divided into low and high-risk groups based on median risk scores. The “dplyr” R package was used to create Sankey diagrams showing cluster distributions with different risk groups and survival outcomes.

### Single-cell distribution analysis

The methodology for examining breast cancer specimens within the single-cell GSE176078 repository involved the following steps. The study capitalized on the meticulous cell annotation provided by the data providers for the initial sequencing data, employing tSNE and UMAP techniques to graphically depict the varied cellular clusters as outlined in the pre-existing labels. Subsequently, the distribution of immune-centric prognostic gene expression across these clusters was illustrated through violin graphs. To discern developmental relationships and lineage differentiation among the diverse cellular subsets, we engaged the “Monocle 2R” software suite. For the subsequent pseudotime trajectory analysis, we isolated half of the T cell population. A random subset of these cells, characterized by an average gene expression above 0.1 and an empirical dispersion value exceeding the threshold of 1 × dispersion fit, was selected. The dimensionality of this cellular subset was then condensed using the “DDRTree” approach, while the “reduceDimension” function served to categorize the various cell differentiation stages. Ultimately, the “plot_cell_trajectory” function was deployed to graphically represent the differentiation pathway of T cells, incorporating the levels of immune-relevant prognostic gene expression throughout their developmental progression.

### Tumor immune and cancer stem cell (CSC) index analysis

The CIBERSORT algorithm was employed to assess the level of immune cell infiltration in the samples. We first evaluated 22 tumor-infiltrating immune cell characteristics to assess the TME infiltration status. Then, the TME scores, including matrix, immune, and estimate scores for high and low-risk groups, were calculated using the “Estimate” R package. Additionally, we explored the relationship between risk scores and stemness scores.

### Tumor mutation and drug sensitivity analysis

The present investigation entailed the extraction of somatic mutational data from the TCGA repository, which was subsequently converted into the mutation annotation format (MAF) using the “maftools” R package. This facilitated the examination of the mutational landscape across samples segregated into high and low-risk cohorts. Furthermore, we quantified the tumor mutation burden (TMB) scores for these two risk stratifications and probed the potential correlation between TMB scores and the corresponding risk scores. Ultimately, leveraging the “pRophetic” R package, we computed the half-maximal inhibitory concentration (IC50) values for commonly employed chemotherapeutic agents in the management of breast cancer. This enabled us to juxtapose the differential therapeutic responses to these chemotherapies between the high and low-risk patient populations.

### RT-qPCR

Total RNA was extracted from normal mammary epithelial cells (MCF-10A) and BC cells (HCC-1954, SUM 159, T-47D, MDA-MB-231), using RNA fast 200 RNA Extraction kit (Fastagen Biotech, Shanghai, 220,010). cDNA was synthesized using the StarScript III RT MasterMix (Genestar). mRNA expression levels were quantified using SYBR-Green assays (Abclonal) in a Bio-Rad CFX-96 instrument (Hercules). Data were processed using the △△CT method, with GAPDH selected as the internal reference. Primers used in this study are listed in Table S3.

### Statistical analysis

All statistical analyses were carried out using R version 4.4.0 software (http://www.r-project.org), employing a two-tailed P-value threshold of less than 0.05 to denote statistical significance.

### Ethics approval and consent to participate

Since the TCGA and GEO database is available to global users, for this type of study formal consent is not required. Therefore, the Ethics Committee of the First Affiliated Hospital of Xi'an Jiaotong University is exempted from review.

## Results

### The m6A regulator landscape in breast cancer

**Figure 2:**
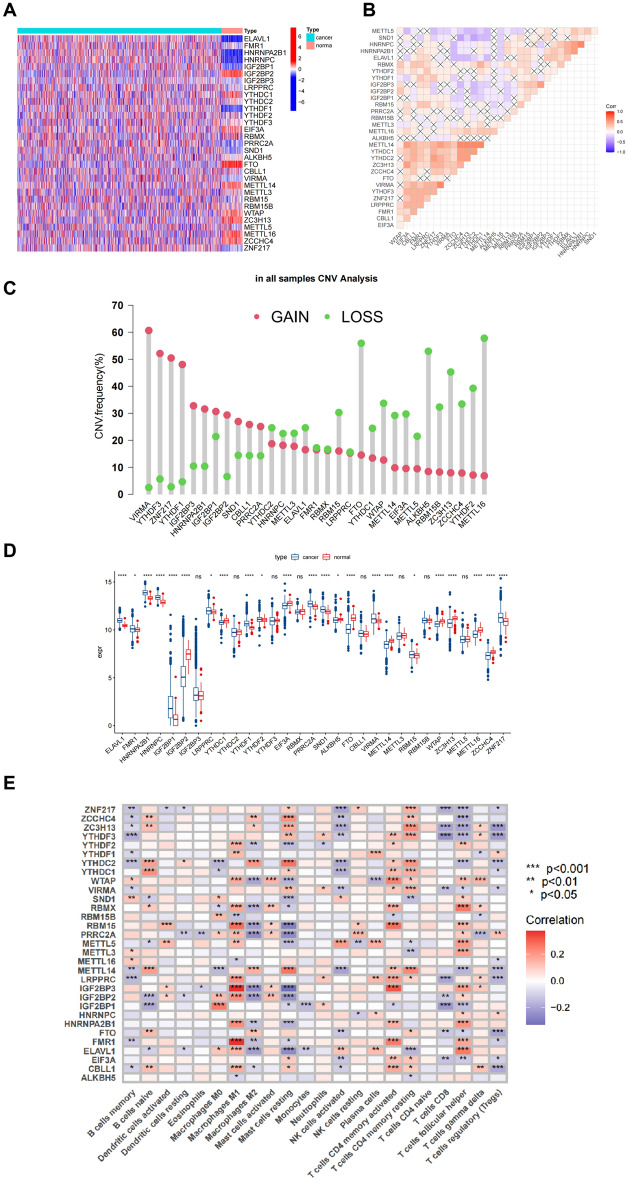
The m6A Regulator Landscape in Breast Cancer (**A**) Comparison of M6A regulatory gene between tumor and normal group. (**B**) Correlation analysis of M6A regulatory genes. (**C**) Frequencies of CNV gain and loss among M6A regulatory genes. (**D**) Expression differences of M6A regulatory genes in tumor tissues and normal tissues. (**E**) Association between the abundance of immune cells and M6A regulatory factors (t-test, ****P < 0.0001; ***P < 0.001; **P < 0.01; *P < 0.05).

In this study, we incorporated 31 m6A RNA methylation regulating genes into the TCGA-BRCA cohort^25,34,35^. Firstly, a heatmap was used to compare the expression differences of these 31 genes in BC and normal tissues (Fig. 2A), revealing inconsistent expression of m6A regulatory factors between tumor and normal tissues. This was followed by a correlation analysis of these 31 genes (Fig. 2B). A summary analysis of somatic mutations in these 31 genes indicated that among 991 BC samples, 78 (7.87%) had mutations in m6A regulatory genes (Fig.s S1A, B), including genes such as FMR1, LRPPRC, YTHDF3, RBM15, WTAP, ZC3H13, RNPA2B1, YTHDC1, YTHDF1, ELAVL1, and IGF2BP2, with missense mutations being the most common. Moreover, we studied the CNVs in these genes, finding that copy number changes are prevalent across all 31 m6A regulatory genes. Specifically, VIRMA, YTHDF3, ZNF217, and YTHDF1 showed extensive CNV gains, while METTL16, FTO, ALKBH5, and ZC3H13 exhibited CNV losses (Fig. 2C, Table S4). The CNV changes of these m6A regulatory genes and their chromosomal positions are shown in Fig. S1C and Table S5. Next, we analyzed the mRNA expression differences of m6A regulatory genes in tumor and normal tissues, as well as in different stages of tumors (Fig. 2D), with ELAVL1, FMR1, HNRNPA2B1, IGF2BP3, YTHDC2, YTHDF2, EIF3A, RBMX, RBM15, and WTAP showing stage-specific expression (Fig. S1D). These results suggest that m6A plays a crucial role in BC. Additionally, we observed that most high-expressed m6A regulatory factors in tumor tissues had CNV gains, such as VIRMA, ZNF217, and YTHDF1. However, some genes like YTHDF3, did not show significant differences in tumor and normal tissues, indicating that CNVs can influence m6A regulatory factors expression, however, it is not the sole determining factor.

Previous studies suggest that the expression of m6A regulators is associated with the heterogeneity of the TME. Therefore, we further analyzed the relationship between m6A regulatory factors and cell infiltration in the TME. Using the CIBERSORT algorithm, we examined the associations between 22 types of immune cells and m6A regulators (Fig. 2E). The heatmap revealed significant enrichments in most of the immune cells. Levels of infiltration of cells such as M1 macrophages and activated memory CD4 T cells were found to be significantly positively correlated with the expression of most m6A regulators. Conversely, infiltration levels of Treg cells and M2 macrophages were significantly negatively correlated with the expression of most m6A regulators. These results indicate that the expression of m6A regulators is significantly correlated with the level of immune cell infiltration, playing an indispensable role in the regulation of the breast cancer TME.

### Identification of m6A subgroup in breast cancer

**Figure 3:**
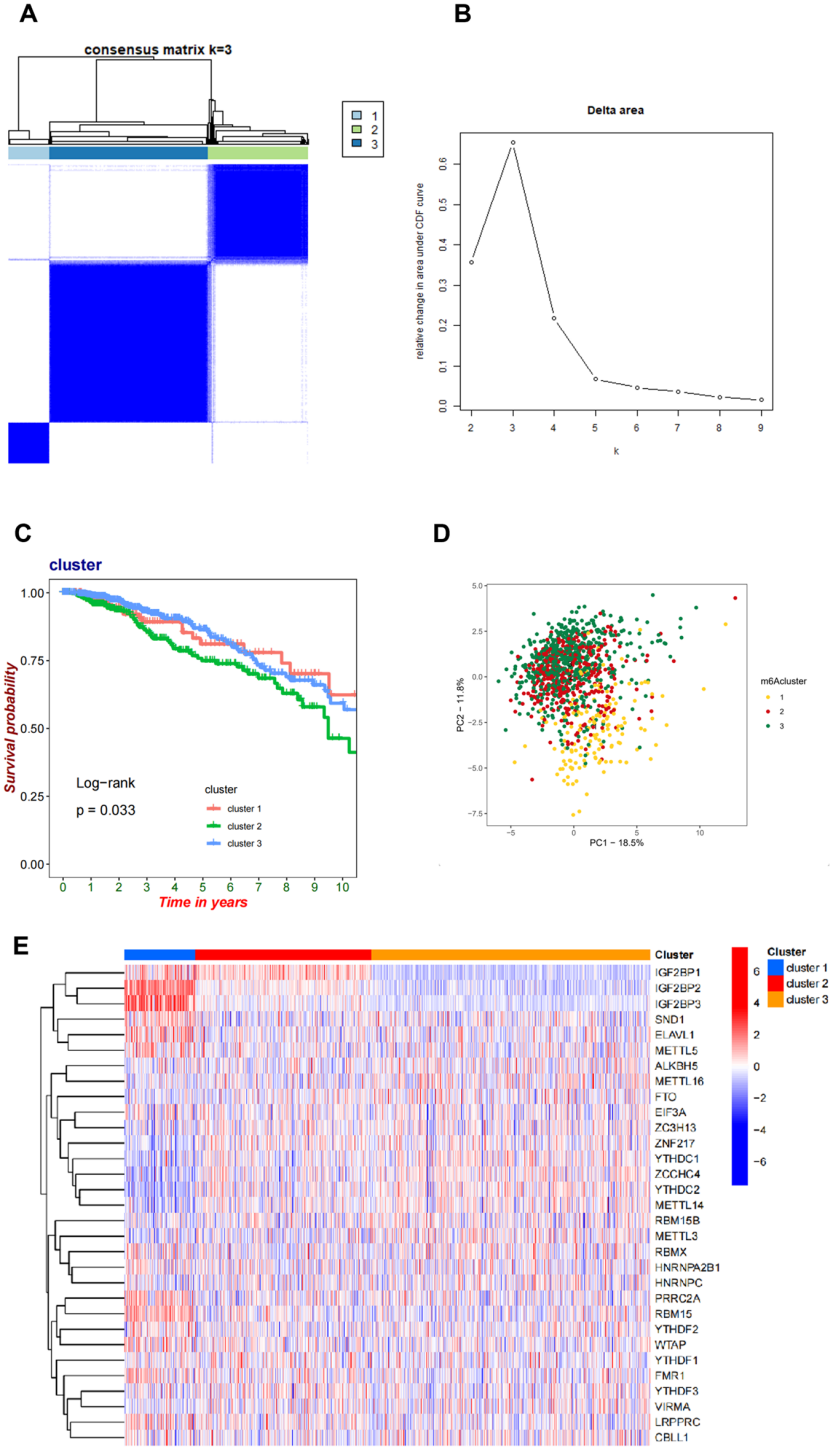
Identification of m6A Subgroup in Breast Cancer. (**A**, **B**) A consensus matrix heat map defining three M6a related subtypes (k = 3). (**C**) Kaplan–Meier analysis of three subtypes of OS. (**D**) PCA analysis of three M6A subtypes. (**E**) The gene expression level of three M6A subtypes.

To study the expression of m6A regulatory genes in BC patients, we employed a consensus clustering algorithm, classifying samples based on the expression patterns of 31 genes. The results showed that when applying k-means clustering with k = 3, patients could be divided into three distinct clusters with strong stability (Fig. 3A-B). Kaplan–Meier survival analysis of the m6A sub-clusters revealed that Cluster 2 exhibited worse prognosis compared to the other two subgroups (log-rank test, P = 0.033; Fig. 3C). Additionally, PCA analysis indicated differences in transcription profiles among the three subgroups (Fig. 3D, Fig. S2A-B). Heatmaps and box diagram were used to compare the m6A regulatory genes distribution of different subgroups of BC (Fig. 3E, Fig. S2C).

### Characteristics of the biological behavior in m6A subgroups

**Figure 4:**
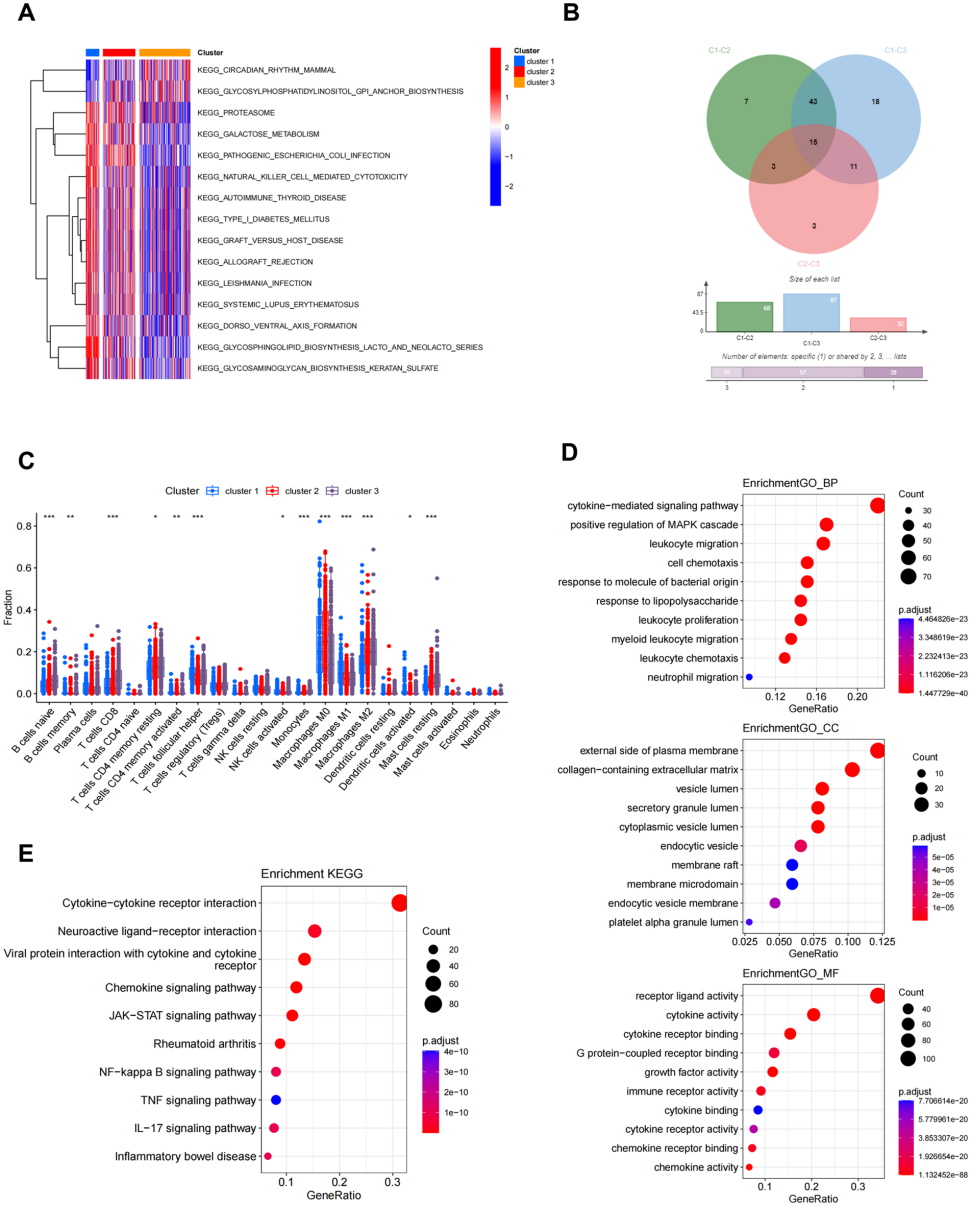
Characteristics of the Biological Behavior in m6A Subgroups. (**A**) GSVA analysis of three M6A subtypes. (**B**) Wayne diagram of three subtypes of GSVA analysis. (**C**) Immune cell infiltration of three M6A subtypes. (**D**, **E**) GO (**D**) and KEGG (**E**) enrichment analysis of DEGs among three M6A subtypes.

GSVA analysis revealed significant statistical differences in the main biological processes enriched in the three subtypes. We first performed pairwise comparisons among these three subgroups, and then intersected the results, yielding a total of 15 biological processes (Fig. 4A-B, Fig. S3A-C). The comparison of each group is shown in Table S6-S8. As shown in Fig. 4A, cluster A was significantly enriched in biological processes like proteasome, glycosphingolipid biosynthesis, etc. Cluster B was mainly enriched in pathways pathogenic escherichia coli infection. Cluster C was enriched in pathways like circadian rhythm. Moreover, the ssGSEA algorithm showed the levels of infiltration of 22 immune cells in the three clusters (Fig. 4C, Fig. S3D). We found statistically significant differences in the infiltration of most immune cells between the three subgroups, such as B cells naive, macrophages, Treg cells and so on. Then, we screened the DEGs related to the subgroup of M6A regulatory gene and crossed it with the previous immune-related genes, and then obtained 321 immune-m6A related DEGs (Table S9). Then we conducted functional enrichment analysis (Fig. 4D, E) based on these DEGs. GO analysis revealed that these immune-m6A related DEGs are involved in biological processes like cytokine-mediated signaling pathway, external side of plasma membrane, receptor ligand activity etc. KEGG pathway analysis shows that these immune-m6A related DEGs are mainly related to cytokine-cytokine receptor interaction, neuroactive ligand-receptor interaction, viral protein interaction with cytokine and cytokine receptor, chemokine signaling pathway, JAK-STAT signaling pathway and so on.

### Construction of m6A-immune-related prognostic risk score

**Figure 5:**
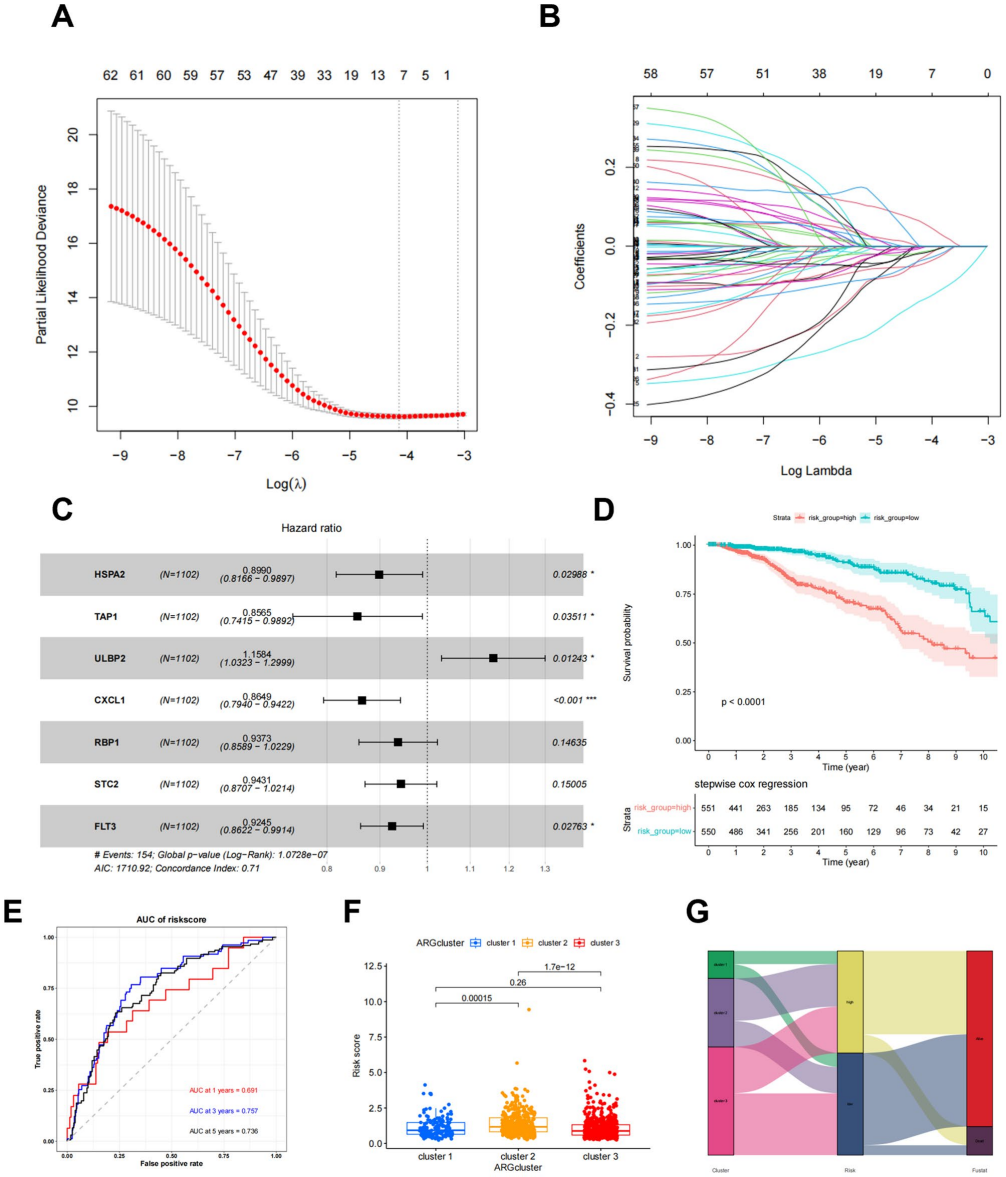
Construction of m6A-Immune-Related Prognostic Risk Score. (**A**, **B**) Lasso regression analysis on the prognosis-related genes. (**C**) multivariate Cox regression analysis. (**D**) OS analysis of two risk groups using Kaplan–Meier. (**E**) ROC curves to predict 1, 3, and 5-year OS according to the risk score in the training cohort. (**F**) The difference of risk score among three m6A gene subtypes. (**G**) The sankey diagram of the sample distribution of three gene subtypes and two risk score groups.

After obtaining 321 m6A-immune-related genes, we then conducted univariate Cox regression on these m6A-immune-related genes to analyze their prognostic value for BC, identifying 62 genes for further analysis (Table S10). Based on these prognosis-related genes, we selected the most prognostic factors through Lasso regression and multivariate Cox regression analysis. According to Lasso regression, 7 OS-related genes (HSPA2, TAP1, ULBP2, CXCL1, RBP1, STC2, and FLT3) were retained through the minimum partial likelihood deviation (Fig. 5A-B and Table S11). Stepwise multivariate Cox regression analysis of these OS-related genes finally led to the construction of a risk model (Fig. 5C). Patients were divided into low-risk (n = 551) and high-risk groups (n = 551) based on median risk score (Table S12). Kaplan–Meier survival analysis of the two risk groups showed that the OS of patients in the low-risk group was significantly better than that in the high-risk group (log-rank test, P < 0.001; Fig. 5D). The 1-year, 3-year, and 5-year AUC values of the risk score model in the training cohort were 0.691, 0.757, 0.736, respectively (Fig. 5E). We also found significant differences in risk score among the initial three m6A subtypes (Fig. 5F). Cluster 2 scored the highest, followed by Cluster 1, with Cluster 3 scoring the lowest. And heat map was used to compare the distribution of these seven genes in two risk groups (Fig. S4A). The Sanji diagram shows the distribution of samples in 3 subgroups of m6A gene and 2 risk score groups (Fig. 5G). At the same time, we also compared the survival differences in the verification set, and the OS of the low-risk group was significantly lower than that of the high-risk group (Fig. S4B, Table S13).

### Development and validation of a prognostic nomogram for breast cancer

**Figure 6:**
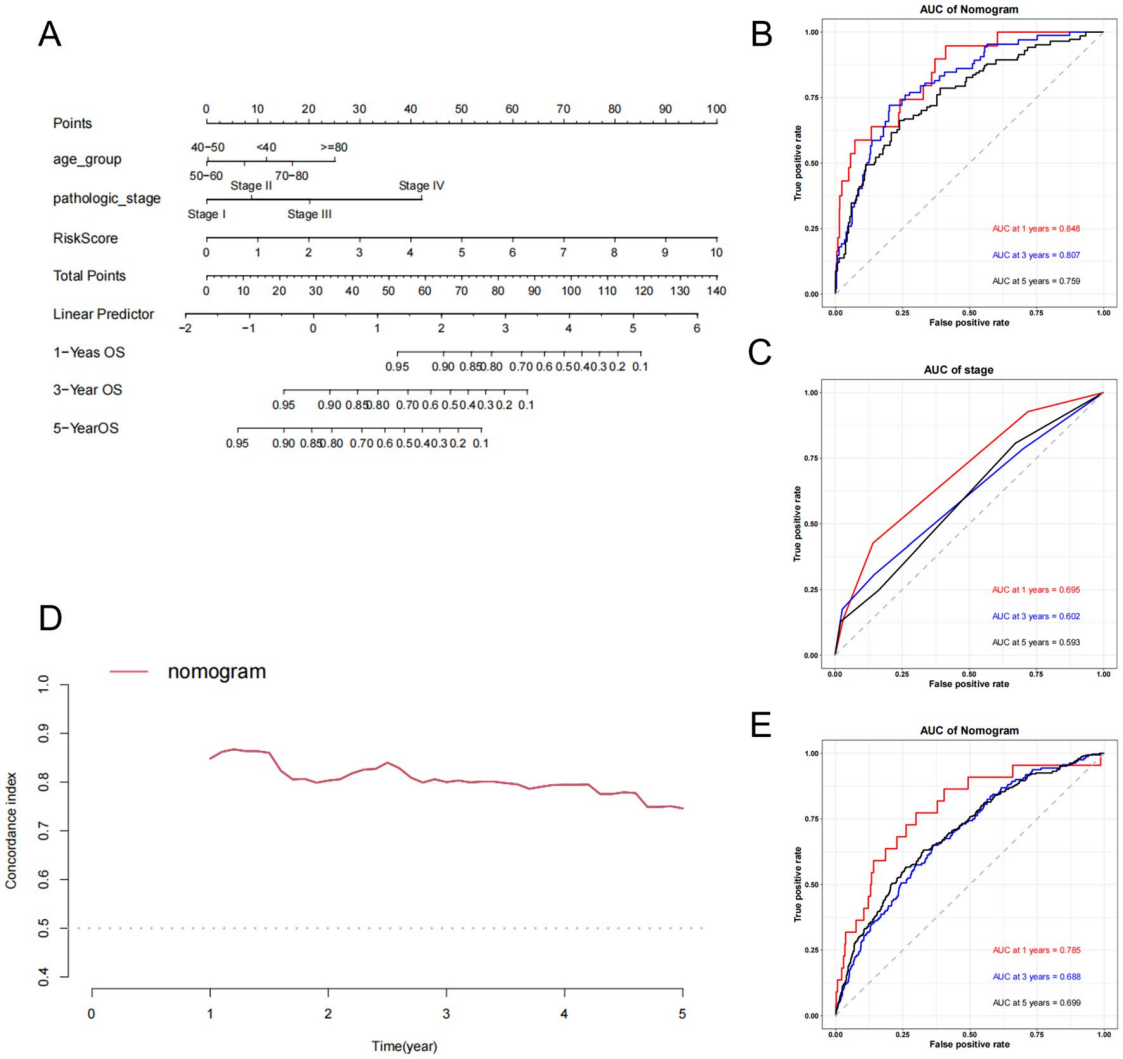
Development and Validation of a Prognostic Nomogram for Breast Cancer. (**A**) A nomogram used to predict BC OS. (**B**) ROC curves to predict 1-, 3-, and 5year OS according to the nomogram in the training cohort. (**C**) ROC curves when clinical indicators are used alone. (**D**) The C-index of the nomogram. (**E**) ROC curves to predict 1-, 3-, and 5year OS according to the nomogram in the testing cohort.

To enhance clinical utility and increase the accuracy of the predictive model, clinical-pathological parameters including age and stage were added to the aforementioned prognostic risk model to construct a more comprehensive nomogram to predict the OS in BC (Fig. 6A). The model was validated to have good discriminative ability. In the training set, the 1-year, 3-year, and 5-year AUC values were 0.848, 0.807, and 0.759, respectively (Fig. 6B), significantly higher than using clinical-pathological parameters alone (0.695, 0.602, 0.593) (Fig. 6C). The C-index curve shows that the model has good consistency (Fig. 6D). In the validation set, the 1-year, 3-year, and 5-year AUC values were 0.785, 0.688, and 0.699, respectively (Fig. 6E).

### Characteristics of the TME, mutation, CSC index, and drug susceptibility analysis in the high and low risk groups

**Figure 7:**
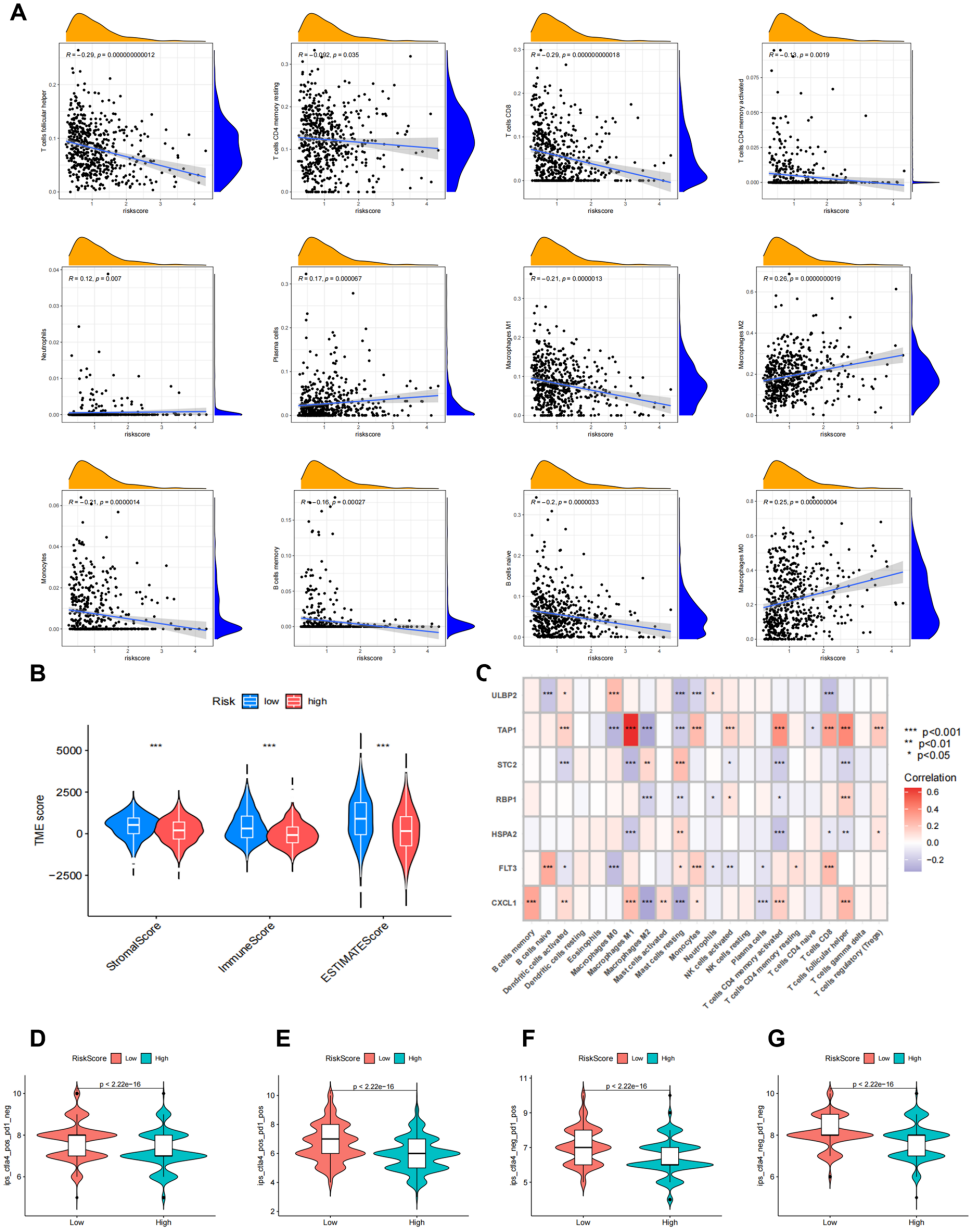
TME and immune checkpoint characteristics in both risk score groups. (**A**) Association of risk score with immune cell infiltration. (**B**) Association between risk score and TME score. (**C**) Association between immune cell infiltration and seven genes in the risk_score model. (**D**–**G**) Immunotherapy effect in the low- and low-risk groups.

The CIBERSORT algorithm was used to assess the correlation between risk score and immune cell infiltration. As shown in Fig. 7A, risk score was negatively correlated with cells such as M1 macrophages and positively correlated with cells such as M2 macrophages. Additionally, compared to a high-risk score, a low-risk score was also associated with a higher immune score (Fig. 7B). We then studied the relationship of the seven genes in the model with immune cells, finding these genes to be significantly related to most immune cells (Fig. 7C). We also observed the differences in immunotherapy between the two risk groups. As shown in the results of Fig. 7D-G, immunotherapy was different between the two groups, and immunotherapy was more effective in the low-risk group.

**Figure 8:**
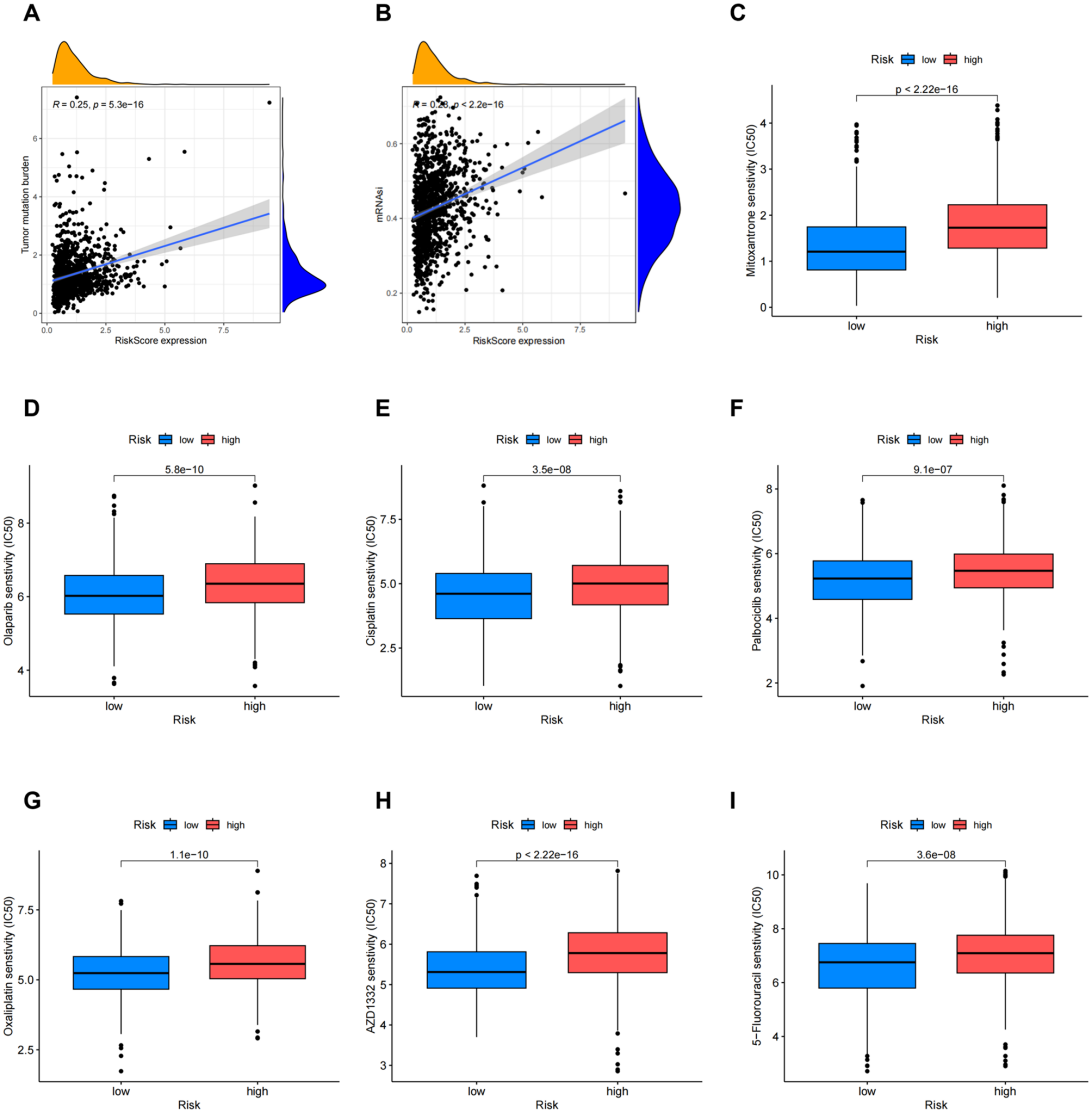
TMB, CSC index and drug susceptibility analysis among two risk_score groups. (**A**) Correlation between risk score and TMB. (**B**) Correlation between risk score and CSC index. (**C**–**I**) Correlation between risk score and drug susceptibility. CSC, cancer stem cells; TMB, tumor mutational burden.

Then we found that there was a positive correlation between Risk Score and TMB (Fig. 8A). Additionally, the potential correlation between the CSC index values and risk score was assessed. According to Fig. 8B, risk score was positively related to the CSC index, suggesting that BC cells with a higher risk score might have more pronounced stem cell characteristics. Finally, we evaluated the susceptibility of patients in high and low-risk groups with BC to some common therapeutic drugs. As shown in Fig. 8C-I. At present, most of the therapeutic drugs have low IC50 in the low-risk group, such as cisplatin, palbociclib, olaparib and 5-fluorouracil.

### Single cell verification of the distribution of m6A-immune prognostic genes in breast cancer

**Figure 9:**
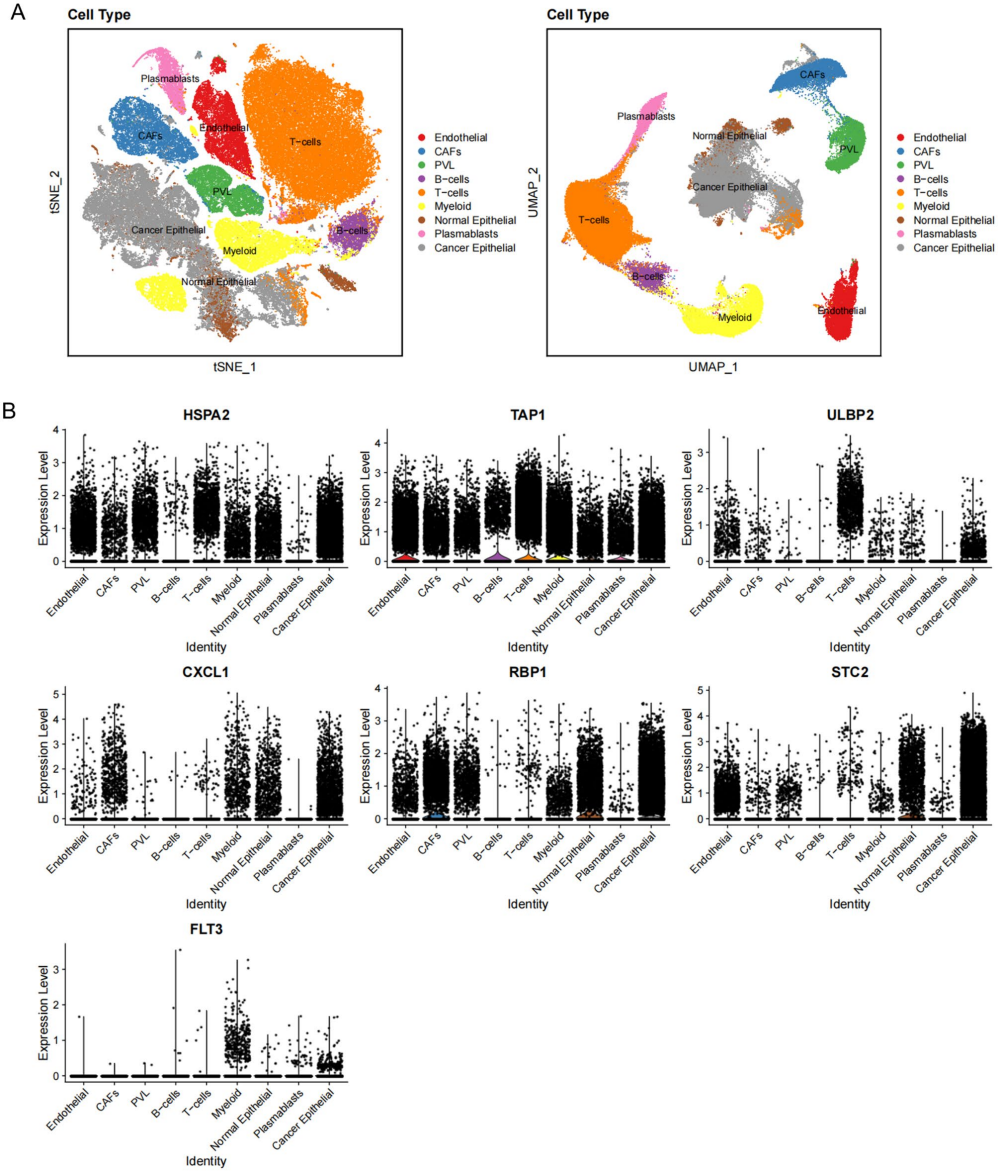
Single Cell Verification of the Distribution of m6A-Immune Prognostic Genes in Breast Cancer. (**A**) tSNE and UMAP projections of breast cancer cells in GSE176078. Different cell types are indicated by unique colors. (**B**) Delineating the distribution of key genes in cell subsets.

**Figure 10:**
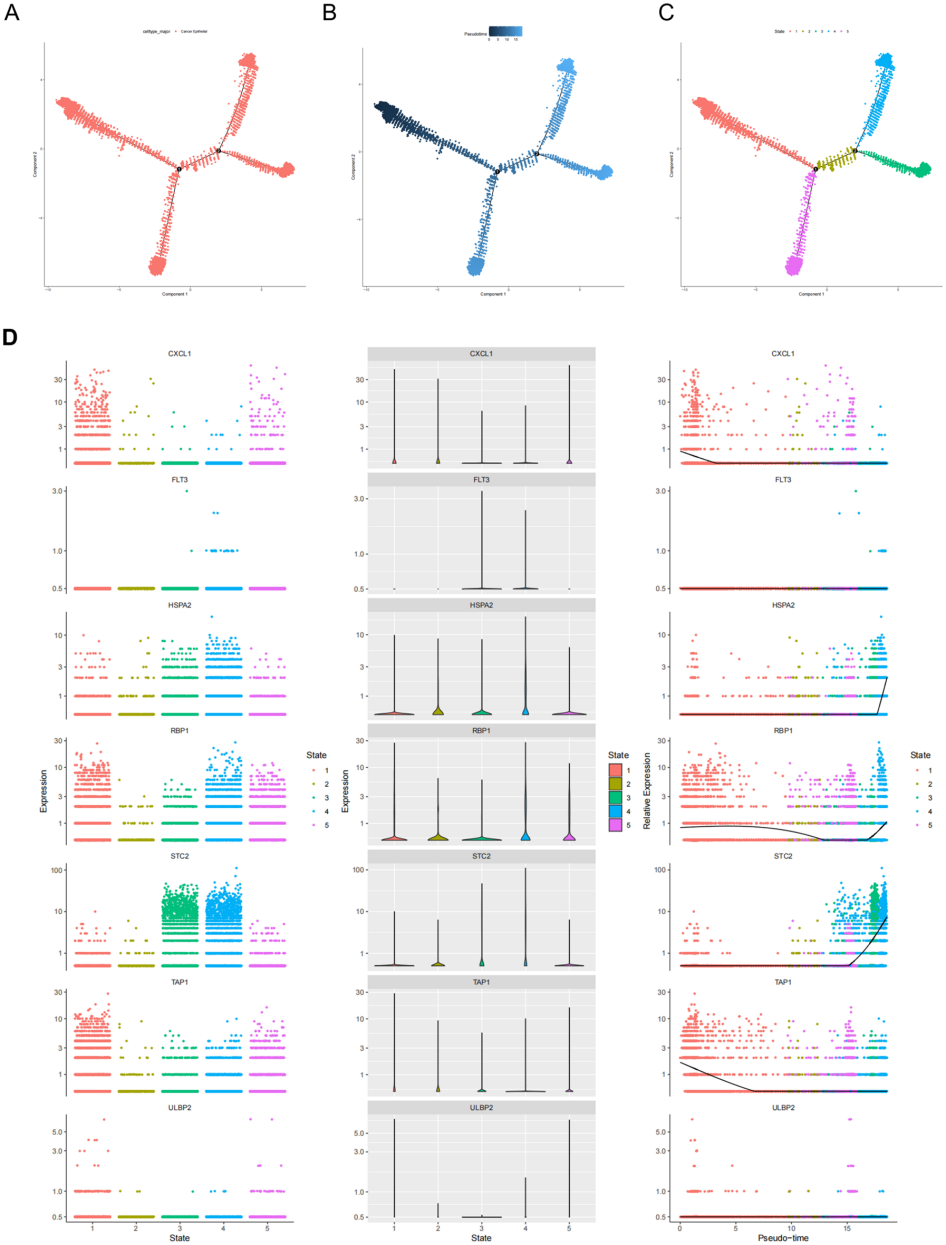
Pseudotime analysis of T cells in triple-negative breast cancer samples from GSE176078. (**A**) All cells analyzed were T cells. (**B**) Differences in the timing of T cell differentiation. The darker blue represents the earlier differentiation stage, while the lighter blue represents the later differentiation stage. (**C**) Five stages of T cell differentiation. State 1 is the earliest stage of differentiation. (**D**) Expression levels of key genes at different stages of T cell development.

Based on the cell annotation results provided by the data provider, cells within BC tissues were categorized into nine cell subpopulations: endothelial cells, cancer-associated fibroblasts (CAFs), perivascular-like (PVL) cells, B cells, T cells, myeloid cells, normal epithelial cells, plasmablasts, and cancer epithelial cells (Fig. 9A). HSPA2 was expressed in most cell clusters, with minimal expression in B cells and plasmablasts. TAP1 was abundantly expressed across all cell clusters, ULBP2 was primarily expressed in T cells, CXCL1 was mainly expressed in CAFs, myeloid cells, normal epithelial cells, and cancer epithelial cells. RBP1 was predominantly expressed in endothelial cells, CAFs, PVL cells, myeloid cells, normal epithelial cells, and cancer epithelial cells. STC2 expression was mainly observed in endothelial cells, normal epithelial cells, and cancer epithelial cells, while FLT3 was primarily expressed in myeloid cells and cancer epithelial cells (Fig. 9B). Subsequently, the cellular trajectory differentiation of T cells was simulated and analyzed (Fig. 10A). The deeper the shade of blue, the earlier the stage of cell differentiation, signifying that T cells undergo differentiation from left to right chronologically, with the darkest blue indicating the earliest differentiated cells. (Fig. 10B). As shown in Fig. 10C, T cells displayed various stages of differentiation, distinguished by distinct colors, with red representing the initial stage of differentiation. The expression levels of HSPA2, TAP1, ULBP2, CXCL1, RBP1, STC2, and FLT3 in the single-cell developmental trajectory were then generated (Fig. 10D), indicating that the expression of CXCL1 and TAP1 was upregulated in early T cell differentiation, while HSPA2 and STC2 expression was upregulated in late T cell differentiation. RBP1 expression was initially downregulated then upregulated during T cell differentiation. Other genes did not show significant changes during T cell differentiation.

### Verification of the expression level of seven m6A-immune related genes in the risk model

**Figure 11:**
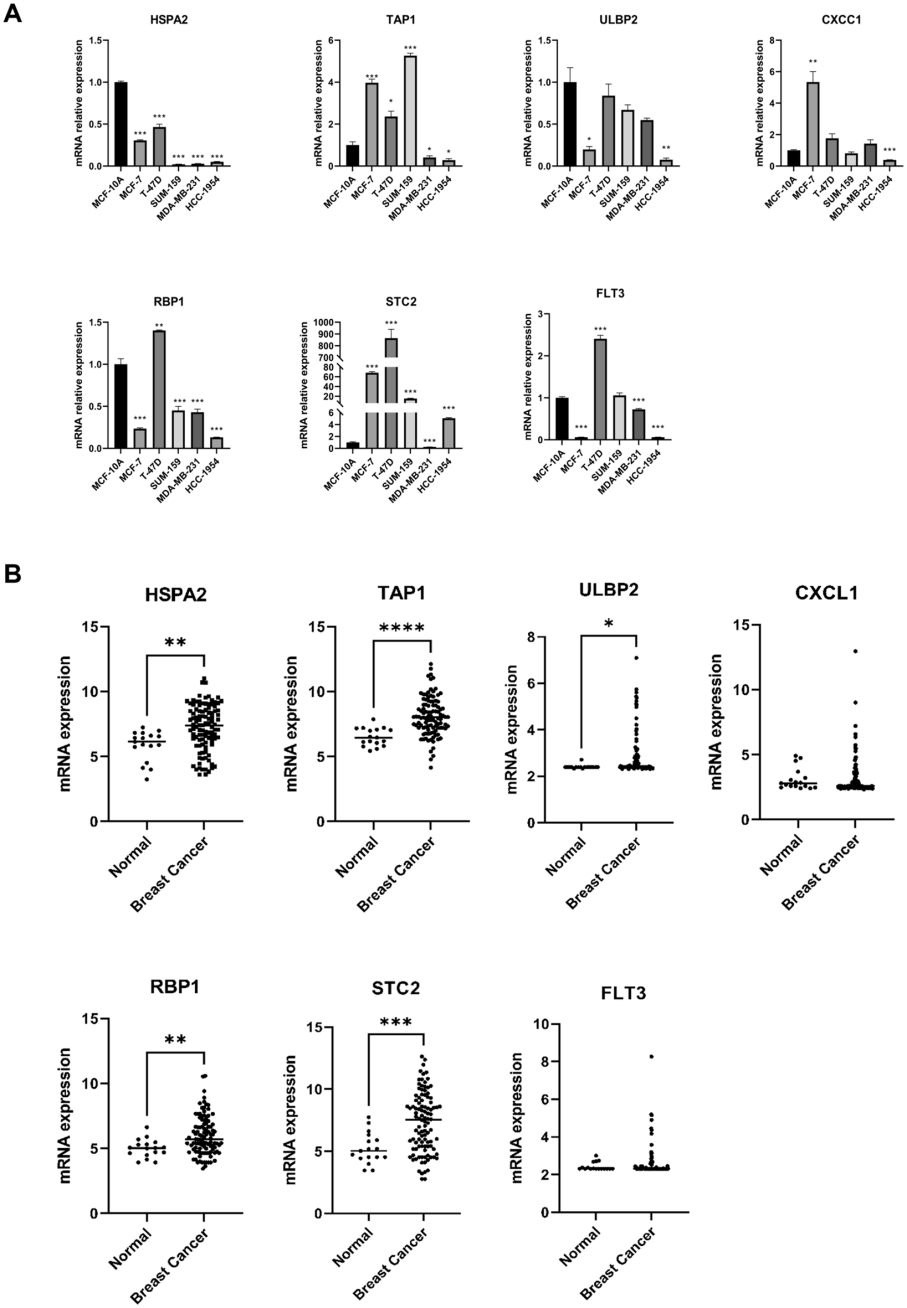
Validation of prognosis-related genes expression. (**A**) RT-PCR was used to detect the mRNA expression of seven prognosis-related M6a-immune genes in breast cancer cells and normal breast epithelial cells. (**B**) mRNA expression of 7 prognostic-related M6a-immune genes from BC patients and corresponding normal tissues (t-test, ***P < 0.001; **P < 0.01; *P < 0.05).

The mRNA expression of seven prognostic genes in the TCGA database is detailed in Table S14. Additionally, we measured the expression levels of these seven prognostic genes in five different BC cell types and one normal mammary epithelial cell type using RT-qPCR. As shown in Fig. 11A, compared to MCF-10A, STC2 expression was significantly upregulated, while the expression levels of HSPA2, ULBP2, and others were downregulated in BC cells. Moreover, the expression levels of these genes in BC patient tissues and corresponding normal tissues were compared using the GSE42568 dataset (Fig. 11B, Table S15), revealing that HSPA2, TAP1, ULBP2, RBP1, and STC2 were significantly upregulated in BC patients, with no marked differences observed for the others.

## Discussion

Despite the advances in cancer research, BC remains a global health challenge. Due to its complexity and diversity, as well as significant individual differences, personalized diagnostics and treatment approaches are required^36–38^. In previous studies, the prognosis of many breast cancer (BC) patients was predicted based solely on clinical information^39^. For example, staging systems are commonly used to predict the prognosis of breast cancer (BC). However, the prognostic ability of a single clinical parameter is limited. Currently, with immunotherapy having become an integral part of cancer treatment, BC exhibits lower immunogenicity and a lower mutational burden compared to other solid tumors. Nevertheless, in some clinical studies, evidence has been observed that supports the synergistic effects of anti-PD1/PD-L1 drugs with chemotherapy^40–42^. In contrast to the rapid development of immune checkpoint inhibitors (ICIs), the development of biomarkers has been relatively slow, with few biomarkers available to continuously assist in the decision-making process for ICIs. N6-methyladenosine, as a major epitranscriptomic modification, contributes to the dynamic regulation of every biological process and has been proven to be closely related to immune dysregulation in the anti-tumor response^43–45^. Therefore, we hope to identify new biomarkers to guide personalized treatment strategies, which can help predict responses to immunotherapy and optimize outcomes for patients with varying risk profiles.

Based on previous literature reports, our study included a total of 31 m6A-related genes. BC samples were classified into three different subgroups based on the differential expression of these genes. We then took the intersection of the DEGs from these three subgroups with immune-related genes. Using LASSO penalized regression and stepwise multiple Cox regression analysis, we identified 7 candidate genes (HSPA2, TAP1, ULBP2, CXCL1, RBP1, STC2, FLT3) as cross genes between the DEGs selected from the training dataset and prognostic-related genes. All these 7 genes showed significant associations with the overall survival in BC.

A brief overview of these seven prognostic genes is as follows. HSPA2, belonging to the HSPA (HSP70) chaperone family, is crucial for male fertility and is found in various somatic organs^46,47^. In BC cases, high levels of HSPA2 are linked to a better prognosis, indicating its potential as a tumor suppressor^48^. TAP1, important in antigen processing and presentation, impacts tumor immunity and is abnormally expressed in several cancer types^49^. Research of Mohammad Sultan highlights that the epigenetic suppression of TAP1 encourages immune evasion and survival in breast cancer stem cells (CSCs)^50^. ULBP2, part of the NKG2D ligands (NKG2DLs) group, shows a connection to BC patient survival and clinicopathological characteristics, with its expression inversely affecting survival rates^51^. The chemokine CXCL1, vital in inflammatory diseases and tumors, shows that its increased presence in BC stroma is associated with higher tumor grade, disease recurrence, and lower patient survival^52,53^. RBP1, an intracellular carrier for retinol and retinaldehyde, is notably reduced in common cancers, including BC^54^. Interestingly, previous studies indicate that STC2 is involved in several malignant tumor processes and is upregulated in various cancers, leading to poorer outcomes^55,56^. Our findings support this, and we found that the expression of STC2 in human breast duct cancer cell line T-47D was about 900 times higher than that in normal breast epithelial cells (Fig. 10), which may offer some insights into the classification and differentiation of breast cancer single cells. Lastly, FLT3, a cell surface receptor tyrosine kinase, plays a role in regulating cell proliferation and survival. Studies suggest that high FLT3 expression in BC is a favorable prognostic indicator, correlating with clinicopathological features and immune infiltration. Our research corroborates these findings, aligning with previous studies and validating the roles of these genes in breast cancer^57^.

This study has developed a model based on the m6A methylation modification within the context of the BC immune microenvironment. Statistically significant differences were observed between low-risk and high-risk score patients in terms of prognosis, TME, TMB, CSC index, and drug sensitivity. Our research results demonstrate that our risk score can be used to evaluate the prognostic significance and immunotherapy response in BC. In our immune analysis, the low-risk group exhibited higher immune scores compared to the high-risk group, suggesting that immunotherapy may be more effective in the low-risk group. Additionally, the risk score was negatively correlated with immune cells such as M2 macrophages and positively correlated with immune cells such as M1 macrophages. Macrophages are the most common TME cell type and play a role in suppressing immune responses within the TME^58^. Inducing macrophage polarization is also a promising cancer treatment strategy^59^. Furthermore, TMB has been previously established as a predictive biomarker for pan-cancer immunotherapy efficacy^20^, and cancer stem cells are also associated with immunotherapy response, targeting CSC may potentially prevent metastasis and improve survival in BC, which we hypothesize might be related to m6A regulation^60^. We believe our research contributes to the precision of breast cancer treatment by identifying patients who are most likely to benefit from immunotherapy, thereby providing more effective personalized treatment plans for these individuals. It can help avoid ineffective treatments for patients who are less likely to benefit from immunotherapy, reducing unnecessary side effects. Additionally, it can assess patients' long-term responses and prognosis to immunotherapy, providing a basis for long-term treatment plans. Despite the slow development of biomarkers related to ICIs in the early stages, which may be due to the complex requirements for tissue samples and sequencing platforms, the rapid advancement of sequencing technology in recent years has greatly deepened our understanding of the differences in gene expression among various types of tumor cells^61^. This will greatly influence our understanding of tumor development, therapeutic strategies, and prognostic assessment. I believe that with the advancement of technology, we will eventually be able to accurately predict the beneficiaries of immunotherapy and provide more effective personalized treatment plans. Cancer, this long-standing adversary to human health, is destined to be ultimately conquered.

Certainly, our study has some limitations. We used public databases to generate model structures and validate results. Due to incomplete clinical data for many patients in the databases, we had to exclude a significant amount of individual data. This may have had a certain impact on the results. Additionally, the lack of single-cell data, as well as limitations in sequencing depth and analysis conditions, prevented more detailed analysis and classification. Most importantly, the absence of a comprehensive clinical study and sequencing database for breast cancer immunotherapy means we cannot directly verify the model's guiding role in immunotherapy. Therefore, conducting prospective studies to assess the clinical application of this model in BC patients is crucial. Finally, the specific mechanisms of action of these genes, their interactions with each other, and possible associations with immune cells require comprehensive functional experiments to elucidate.

## Conclusion

In our current research, we have identified seven novel m6A-immune prognosis-related genes from public databases. We observed that patients with a lower risk had longer survival times. Based on these genes, we constructed a prognostic nomogram that also includes other clinical parameters, such as age and pathological staging. This model demonstrates good predictability and relative stability in accuracy. These findings hold promise for offering new strategies in the personalized treatment of BC and improving clinical benefits.

### Supplementary Information

Supplementary Information 1.

Supplementary Information 2.

## Data Availability

Publicly available datasets were analyzed in this study. This data can be found here: https://portal.gdc.cancer.gov/, https://www.ncbi.nlm.nih.gov/geo/, https://www.cbioportal.org and https://www.cancer.gov/ccg/research/genome-sequencing/tcga.
