# Supplementary material for: Identifying subtypes and developing prognostic models based on N6-methyladenosine and immune microenvironment related genes in breast cancer
Source: Sci Rep. 2024 Jul 18;14:16586. doi: 10.1038/s41598-024-67477-w (PMC11255230; doi:10.1038/s41598-024-67477-w)
Supplement: Supplementary file 1 — Supplementary Information 1. [file 41598_2024_67477_MOESM1_ESM.docx]

**
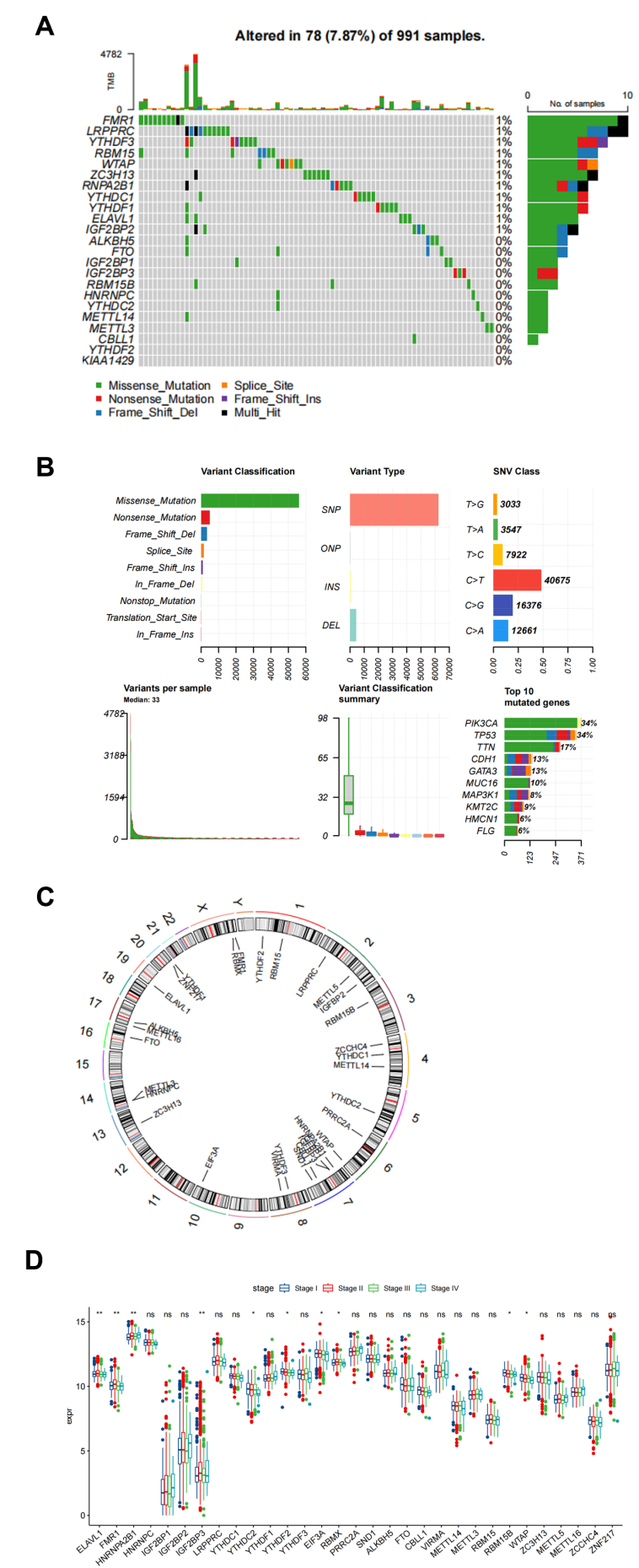
**

**Fig. S1**

**The m6A Regulator Landscape in Breast Cancer** (A, B). Somatic mutations and types of M6A regulatory genes; (C). The expression of M6A regulatory gene in different tumor stages；(D). Location of CNV on 23 chromosomes of M6A regulatory gene.

**
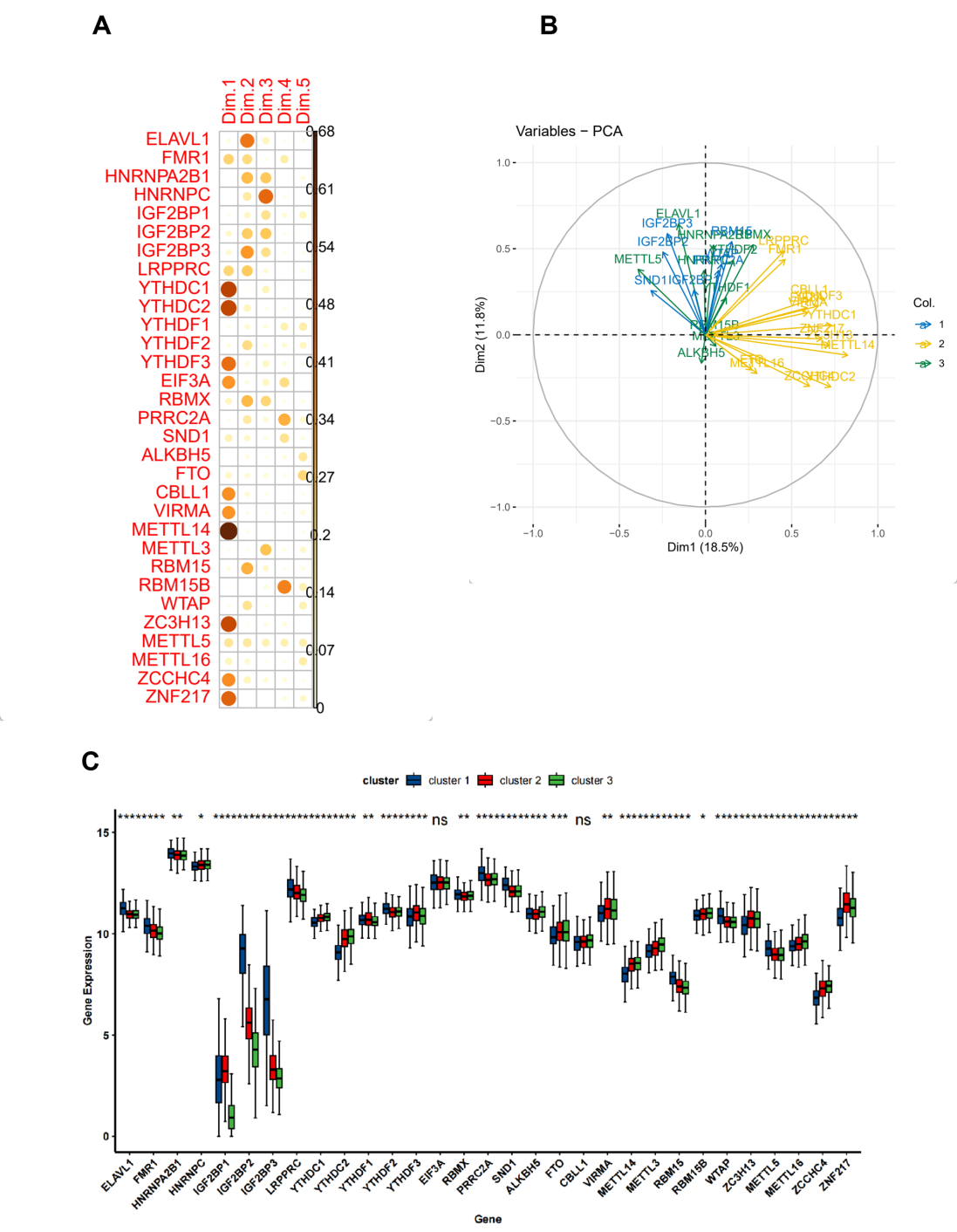
**

**Fig. S2**

**Identification of m6A Subgroup in Breast Cancer** (A, B). PCA analysis of three m6A subtypes; (C). Differences in the expression of m6A regulatory genes in three m6A subtypes.

**
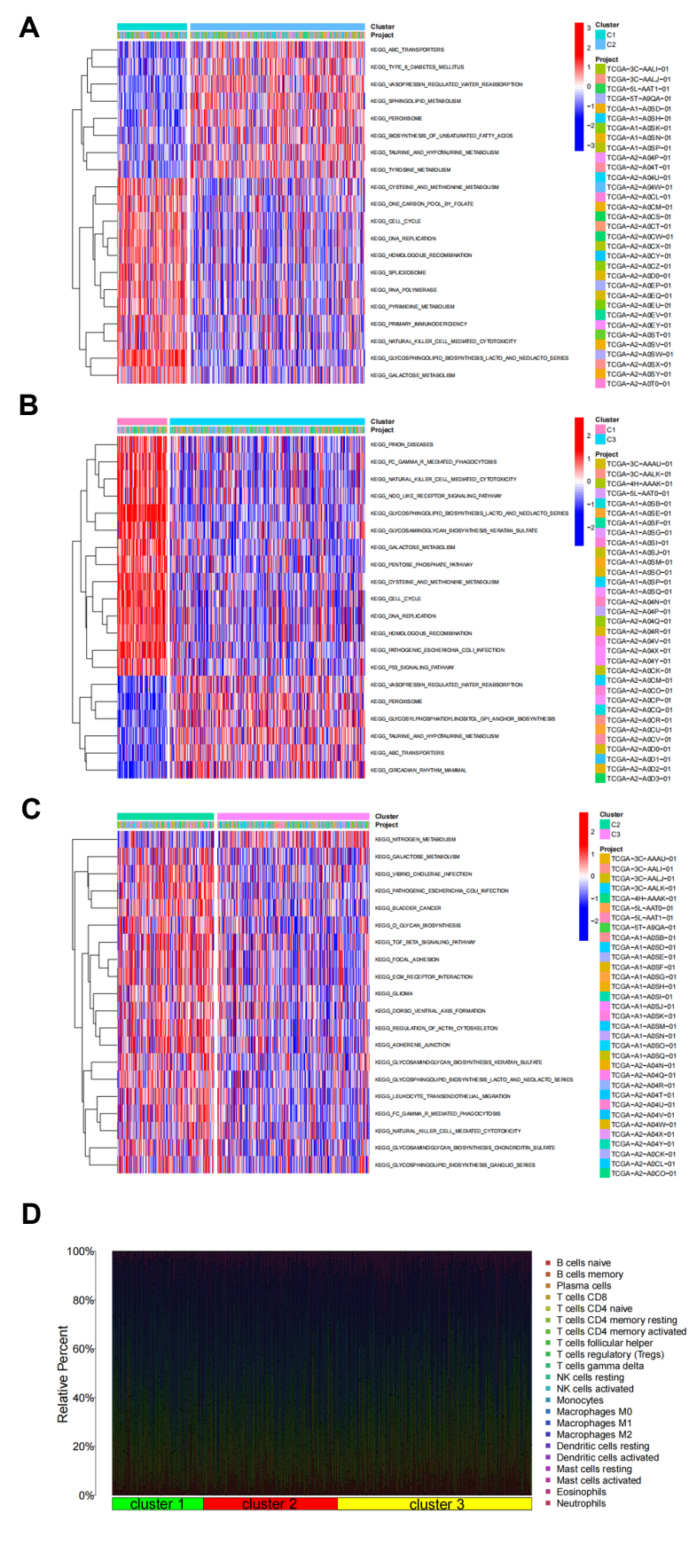
**

**Fig. S3**

**Characteristics of the Biological Behavior in m6A Subgroups** (A-C). GSVA analysis among three M6A subtypes. (D). The proportion of immune cell infiltration of three M6A subtypes.

**
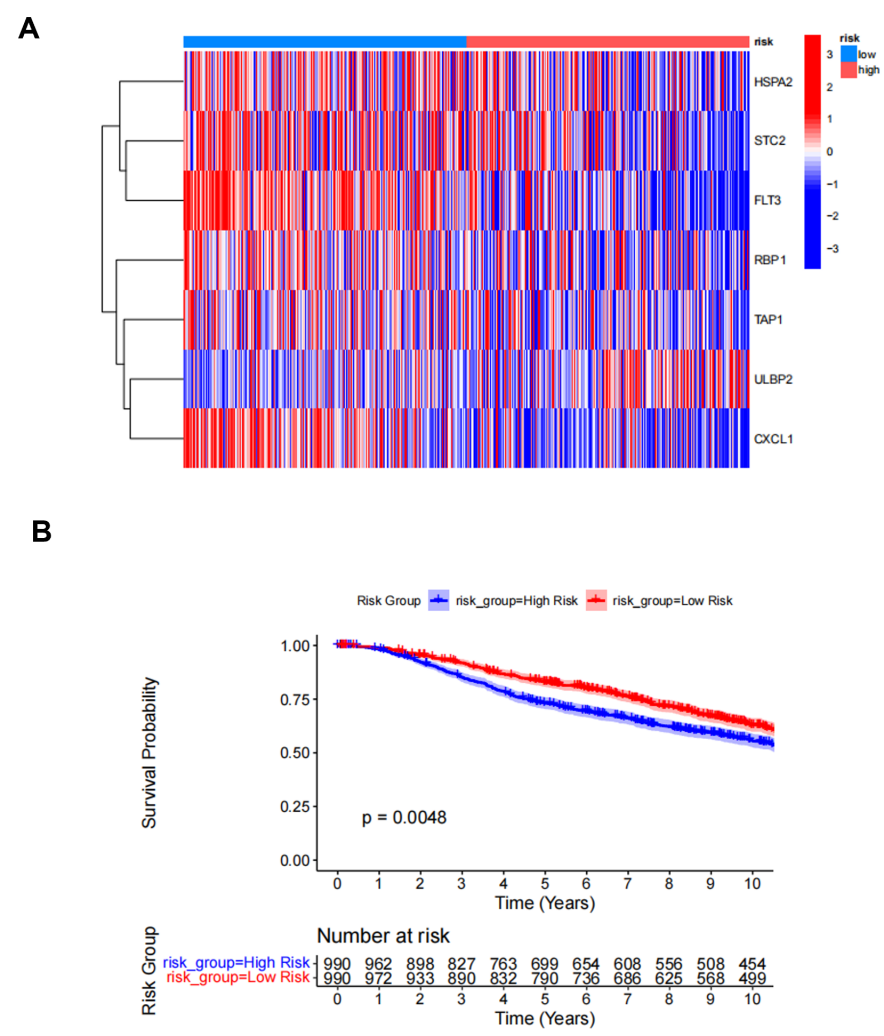
**

**Fig. S4**

**Construction of m6A-Immune-Related Prognostic Risk Score** (A). Differences in gene expression between two risk groups; (B). Kaplan-Meier analysis of OS differences between the two risk groups in the verification group
